# The Protective Effects of Danggui-Baizhu-Tang on High-Fat Diet-Induced Obesity in Mice by Activating Thermogenesis

**DOI:** 10.3389/fphar.2018.01019

**Published:** 2018-09-05

**Authors:** Lijun Zhao, Xiaoqiang Zhu, Renhuai Cong, Xiangliang Yang, Yanhong Zhu

**Affiliations:** ^1^National Engineering Research Center for Nanomedicine, College of Life Science and Technology, Huazhong University of Science and Technology, Wuhan, China; ^2^Joint Laboratory for the Research of Pharmaceutics, Huazhong University of Science and Technology, Wuhan, China

**Keywords:** Danggui-Baizhu-Tang, obesity, high-fat diet, brown adipose tissue, white adipose tissue, thermogenesis

## Abstract

Danggui-Baizhu-Tang (DBT), a traditional Chinese medicine decoction, was used for decreasing serum TG and TC remarkably. However, effect of weight control and action mechanism remains confused. In this study, to evaluate the anti-obesity effects, different gradient concentration of DBT (0.59, 1.17 g/kg) or Orlistat (Orl, 15.6 mg/kg; positive control) were administrated by gavage for 8 weeks in C57BL/6J mice which were pretreated with chow or high fat diet (HFD) for 3 months. After administration, significant decrease of body weight and food utilization was observed. It was indicated that concentration of triacylglycerol (TG), total cholesterol (TC), alanine aminotransferase (ALT), aspartate aminotransferase (AST) in serum were reduced strikingly, as well as accumulation of lipid droplets in liver. Meanwhile, DBT treatment could also decrease weight of white adipose tissue (WAT) and size of adipocytes, whereas increase weight of brown adipose tissue (BAT) in mice. Moreover, it was revealed that DBT could elevate rectal temperature by raising expression of uncoupling protein-1 (UCP1) and peroxisome proliferator-activated receptor-gamma coactivator-1alpha (PGC-1α), which were attributed to phosphorylation of AMP-activated protein kinase (AMPK). Furthermore, TNF-α and IL-6, obesity-related inflammatory cytokines, were decreased. In conclusion, DBT could stimulate phosphorylation of AMPK to raise expression of UCP1 and PGC-1α, and activate thermogenesis to prevent obesity.

## Introduction

Obesity is one of the major challenges for public health in the world, and the prevalence of obesity has augmented dramatically in recent years (Dirinck et al., 2011; Flegal et al., 2012). According to the World Health Organization (WHO) reports, the number of obesity people has quadrupled in the last three decades (Mishra et al., 2016; Pathak et al., 2018). Obesity is the root reason of metabolic syndrome and chronic diseases, such as type 2 diabetes, hypertension and cardiovascular diseases (Haslam and James, 2005; Eckel et al., 2011). Obesity involves communications between organs and tissues, such as adipose tissue and liver (Song et al., 2017). It is highly plausible that enlarged adipose tissue is related to high TG, TC, and free fatty acid, which further influence liver lipid metabolism (Peng et al., 2017).

When energy intake exceeds energy expenditure, obesity develops. Meanwhile, increased lipid droplets in liver and fat accumulation in adipose tissue were observed (Woods et al., 2004). Currently, several drugs such as sibutramine and orlistat approved by Food and Drug Administration were applied for long-term use to prevent obesity (Joyal, 2004; Chen G. et al., 2017). Sibutramine could inhibit re-uptake of monoamine signaling elements, increase energy consumption and reduce fat accumulation (Sibutramine for obesity, 1998). Orlistat, a gastrointestinal lipase inhibitor, reduced the intestinal triglyceride absorption (Hsieh et al., 2005). However, some side effects, limited their clinical application (Colman et al., 2012; Krentz et al., 2016). Therefore, it is urgent to develop safer and more effective strategies (Wu T. et al., 2013). It is known that the aim of traditional Chinese medicine is to achieve harmony and promote moderation, which is different from western medicine. Therefore, natural traditional herbs and their bioactive compounds, such as berberine (Zhang et al., 2014), celastrol (Ma et al., 2015), and cinnamomum cassia (Song et al., 2017), have been researched. Danggui-Baizhu-Tang (DBT), a traditional Chinese medicine decoction, was first described in *Sanyin Ji Yi Bingzheng Fang Lun* in AD 1174. DBT, contains four crude herbs: Danggui (Radix Angelicae Sinensis), Fuling (Poria), Baizhu (Rhizoma Atractylodis Macrocephalae), and Zhishi (Fructus Aurantii Immaturus). Radix Angelicae sinensis could prevent metabolic disorders by maintaining the lipid homeostasis (Hua et al., 2014; Zhong et al., 2017). Poria exerted the effects of lipogenesis inhibition (Wang et al., 2011). Rhizoma Atractylodis Macrocephalae possessed anti-obesity effects through inhibiting lipid accumulation (Kim et al., 2011; Wang et al., 2015). And Fructus Aurantii Immaturus could prevent high-fat diet-induced obesity (Iizuka et al., 1998). It has been proposed that DBT could decrease TG and TC to protect people from alcoholic fatty liver (Hai-xiao et al., 2011). However, the anti-obesity effects of DBT have not been investigated and its action mechanism remains confused.

In this study, we explored the effects of DBT on obese mice induced by HFD. It was revealed that administration of DBT significantly decreased body weights, TG, TC, and lipid droplets in liver. And it also reduced white adipose tissue weight and the size of adipocytes. Moreover, increased expression of UCP1 and PGC-1α through phosphorylation of AMPK could elevate rectal temperature in DBT treated mice.

## Materials and Methods

### Preparation of Danggui-Baizhu-Tang

DBT was provided by Infinitus Company Ltd. (China), and the chemical quality control was shown in **Supplementary Table S1**. Mixture of 3 g Poria, 2 g Radix Angelicae sinensis, 2 g Rhizoma Atractylodis Macrocephalae and 2 g Fructus Aurantii Immaturus was extracted two times with boiling water, one with 90 mL for 1.5 h, another with 72 mL for 1 h. Then the mixture of extracts was concentrated to obtain the decoction.

### Animals and Diets

Fifty C57BL/6J male mice (18–20 g) were purchased from Hubei Center for Disease Control and Prevention, Wuhan, China (quality certification number: SCXK (E) 2015-0018). The mice were housed in cages at 22 ± 2°C, 55 ± 5% relative humidity and with a 12 h light–dark cycle. The mice were given a chow diet (chow, 10%, D12450B, Research Diet, New Brunswick, NJ, United States) or high fat diet (HFD, 60%, D12492, Research Diet, New Brunswick, NJ, United States) until the end of the experiment. And the compositions of diets are shown in **Table 1**. After acclimatization for 1 week, the mice were fed with chow or high-fat diet for 20 weeks. After the first 12 weeks on the high-fat diet, the obesity status was observed and the mice were randomly divided into 4 groups (*n* = 8): HFD group (HFD), DBT-Low group (DBT-L, 0.59 g/kg), DBT-Middle group (DBT-M, 1.17 g/kg) (Wu et al., 2014) and Orlistat group (Orl, 15.6 mg/kg). The mice were administrated with saline, DBT-L, DBT-M or Orl by gavage for the subsequent 8 weeks, respectively. The mice had access to food and water *ad libitum*, body weight and the amount of food intake in each group were recorded weekly. Lee index was calculated as: [body weight (g) × 1,000/naso-anal length (cm)] 1/3 (Hioki et al., 2010). Food efficiency ratio (FER) was calculated as follows: FER (%) = body weight gain (g/d) /food intake (g/d) × 100 (Chen G. et al., 2017). The body temperature of the mice was measured at room temperature at 0900 h using a rectal probe connected to digital thermometer (BAT-12 Microprobe-Thermometer; Physitemp; United States). At the end of the experiment, all mice were fasted overnight and sacrificed via cervical dislocation. Sera were collected and stored at –20°C. Then the liver and adipose tissue were removed, rinsed with a physiological saline solution, weighed, and rapidly stored at –80°C. All animal studies were approved by the Animal Experimentation Ethics Committee of College of Life Science and Technology, Huazhong University of Science and Technology. The animal study was carried out in strict with guidelines approved by the Science and Technology Department of Hubei Province.

**Table 1:** Composition of the diets and energy densities.

Ingredient	Low-fat diet		High-fat diet	
	g%	kal%	g%	kal%
Casein, 30 Mesh	18.96	19.72	25.84	19.72
Corn starch	29.86	31.05	0.00	0.00
Maltodextrin 10	3.32	3.45	16.15	12.34
Sucrose	33.17	34.51	8.89	6.78
Cellulose, BW200	4.74	0.00	6.46	0.00
Soybean oil	2.37	5.55	3.23	5.55
Lard	1.89	4.44	31.66	54.35
Mineral mix S10026	0.95	0.00	1.29	0.00
DiCalcium phosphate	1.23	0.00	1.68	0.00
Calcium carbonate	0.52	0.00	0.71	0.00
Potassium citrate, 1 H20	1.56	0.00	2.13	0.00
Vitamin mix V10001	0.95	0.99	1.29	0.99
Choline bitartrate	0.19	0.00	0.26	0.00
FD&C yellow dye #5	0.005	0.00	0.006	0.00
Total	100	100	100	100
Protein	19.2	20	26.2	20
Carbohyd rate	67.3	70	26.3	20
Fat	4.3	10	34.9	60


### Biochemical Analyses

The blood was collected in a tube and centrifuged at 3,000 g for 10 min. The levels of free fatty acid, TG, TC, ALT, and AST in sera were detected with automatic biochemical analyzer (Beckman, Germany). Serum insulin was measured with INS ELISA kit (Nanjing Jiancheng Institute of Biotechnology, China).

### Oral Glucose Tolerance Test (OGTT)

A glucose tolerance test was performed in mice after an overnight fast. The glucose concentrations were determined with a blood glucose meter (Accu-Check Active 1, Roche Pharmaceutical Ltd., Basel, Switzerland) and measured in blood collected from the tail vein immediately before and, 30, 60, and 120 min, after orally administration of glucose at 2 g/kg (63005518, Sinopharm Chemical Reagent Co., Ltd., China).

### Oil Red O Staining

To examine lipid deposition in liver, tissues were fixed in formaldehyde, embedded, sliced and stained with Oil-Red O and counterstained with hematoxylin. The pictures were taken with a light microscope (Nikon Eclipse TE2000-U, NIKON, Japan).

### Histology and Immunohistochemistry

Tissues were fixed in 4% paraformaldehyde and embedded in paraffin according to standard procedures. Liver and adipose tissue sections of 5 μm thickness were stained with hematoxylin and eosin for general morphological observations. UCP1 (23673-1-AP, 1:100, Proteintech, China) and p-AMPK (#2535, 1:100, Cell Signaling Technology, United States) were stained for immunohistochemistry. The images were acquired using a light microscope (Nikon Eclipse TE2000-U, NIKON, Japan) and quantification with Image-Pro Plus (Media Cybernetics, Rockville, MD, United States).

### Quantitative Real-Time PCR Analysis

Total RNA of liver and adipose tissue was extracted using the RNAiso Plus reagent. Isolated RNA was quantified by measuring OD at 260 and 280 nm with NanoDrop 2000 spectrophotomer (Thermo Fisher Scientific, Waltham, MA, United States). The cDNA was synthesized according to the PrimeScript RT reagent Kit (Takara Biotechnology Co., Ltd., China) and the assay was performed using real-time PCR with SYBR Premix Ex TaqTM I and 7500 Real Time PCR System (Applied Biosystems, United States). Sequences of the primers (Chen G. et al., 2017) used in the study were listed in **Table 2**. The GAPDH gene was selected as the housekeeping gene in our study.

**Table 2:** Primers used for quantitative real-time PCR.

Primer name	Forward	Reverse
UCP1	TACACGGGGACCTACAATGCT	TCGCACAGCTTGGTACGCTT
PGC-1α	TCACGTTCAAGGTCACCCTA	TCTCTCTCTGTTTGGCCCTT
TNF-α	GGAACACGTCGTGGGATAATG	GGCAGACTTTGGATGCTTCTT
IL-6	AGTTGCCTTCTTGGGACTGA	GCCACTCCTTCTGTGACTCC
Fas	GGAGGTGGTGATAGCCGGTAT	TGGGTAATCCATAGAGCCCAG
Srebp-1	GCATGCCATGGGCAAGTAC	CCACATAGATCTCTGCCAGTGTTG
Chrebp	CGGGACATGTTTGATGACTATGTC	CATCCCATTGAAGGATTCAAATAAA
GAPDH	GTTCCTACCCCCAATGTGTCC	TAGCCCAAGATGCCCTTCAGT
BMP4	TTCCTGGTAACCGAATGCTGA	CCTGAATCTCGGCGACTTTTT
Smad1	GCTTCGTGAAGGGTTGGGG	CGGATGAAATAGGATTGTGGGG


### Western Blot

Adipose tissues were lysed in RIPA buffer with protease inhibitors, and centrifuged at 12,000 g for 20 min at 4°C. After quantification with BCA kit, 10 μL protein of each group were electrophoresed on 8% SDS-acrylamide gels. Blots were incubated at 4°C overnight with primary antibodies (1:1,500) against anti-phospho-AMPKα (Thr172), anti- AMPKα, anti-PGC-1α, and anti-GAPDH (#2535, #2532, #2178, Cell Signaling Technology, Danvers, MA United States) respectively. And then washed three times with TBST (0.5% Tween-20 in TBS) and incubated with the secondary antibody (A0208, 1:10,000, Beyotime, China) for 2 h. The images were acquired with ChemiDoc XRS+ (Bio-Rad, United States).

### Statistical Analysis

All the data were given as mean and standard error of mean (SEM). Statistical analysis was conducted by the SPSS 22 software (SPSS Inc., Chicago, IL, United States). The statistical differences between the groups were evaluated by one-way ANOVA with Least Significant Difference (LSD) test. In any case, *p* < 0.05 was considered as statistically significant.

## Results

### DBT Prevents HFD-Induced Obesity

**FIGURE 1:**
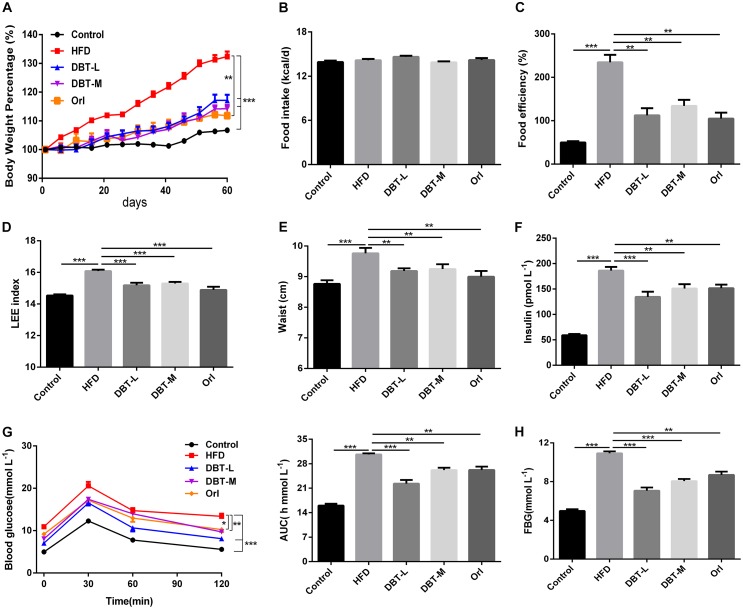
DBT prevents obesity and improves glucose metabolism. **(A)** Body weight percentage of high-fat diet-induced obese mice; **(B)** Food intake of mice per day; **(C)** Food efficiency of mice; **(D)** LEE index; **(E)** Waist circumstance; **(F)** fasting insulin levels; **(G)** Oral glucose tolerance test and the average area under the curve of oral glucose tolerance test; **(H)** Fasting blood glucose levels. Results are presented as mean ± SEM (*n* = 7). ^∗^*p*< 0.05, ^∗∗^*p*< 0.01, ^∗∗∗^*p*< 0.001 compare with HFD group.

To assess whether DBT could prevent HFD to induce obesity, the related physiological and serological indicators were detected. Compared with control group, body weight and food efficiency significantly increased in HFD group, and in contrast, obvious decrease were observed in DBT groups and Orl group (**Figures 1A,C**). Food intake among different groups barely changed (**Figure 1B**), which indicated the decreased body weight was not attributed to food intake. Consistent with these results, Lee index, an evaluation index of obesity (Hioki et al., 2010) was reduced in DBT and Orl treated groups compared with that in the HFD group (**Figure 1D**), as well as waist circumference (**Figure 1E**). Besides, serum insulin was also significantly reduced in the DBT and Orl treated groups compared with the HFD group (**Figure 1F**). To evaluate the effect of DBT on glucose tolerance induced by obesity, OGTT was conducted. Administration of DBT and Orl in mice resulted in a lower fasting blood glucose (FBG) (**Figure 1H**) and all had a better tolerance to glucose load in comparison with the HFD group (**Figure 1G**), especially in the DBT-L group. These results showed that DBT can prevent obesity and improve glucose metabolism.

### DBT Reduces Hepatic Fat Deposition

**FIGURE 2:**
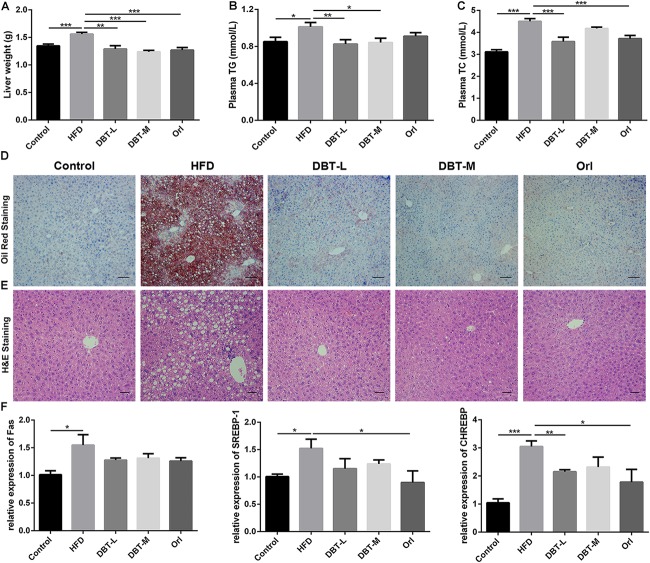
DBT decreases liver lipid accumulation. **(A)** Liver weight; **(B)** Serum TG; **(C)** Serum TC; **(D)** Oil Red staining of liver, scale bar: 50 μm; **(E)** Haematoxylin and eosin staining of liver, scale bar: 20 μm; **(F)** qPCR assays of the liver mRNA levels of Fas, SREBP-1, CHREBP. Results are presented as mean ± SEM (*n* = 7). ^∗^*p*< 0.05, ^∗∗^*p*< 0.01, ^∗∗∗^*p*< 0.001 compare with HFD group.

Obesity has been implicated in the metabolic syndromes including liver diseases, such as non-alcoholic fatty liver disease and non-alcoholic steatohepatitis (Diehl, 2010; Sun and Karin, 2012), so we evaluated whether DBT could decrease liver lipid accumulation. Liver weight in HFD group was heavier than that in the DBT and Orl groups (**Figure 2A**). Compared with DBT and Orl group, more fat deposition in liver tissues in the HFD group were observed by means of Oil Red O staining (**Figure 2D**). Besides, the histomorphological status of the liver cells in DBT and Orl treated groups was similar to the cells in the control group. However, fat cavitation was significantly increased in liver cells of HFD group (**Figure 2E**). In line with these results, lower plasma TG and TC levels were confirmed in the DBT and Orl treated groups than that in the HFD group (**Figures 2B,C**), especially in the DBT-L group. And qRT-PCR assays of liver RNAs also confirmed that DBT and Orl treated groups could decrease the expression levels of lipogenesis factors, such as Fas, CHREBP and SREBP-1 (**Figure 2F**). In addition, obesity accompanied by chronic inflammation was also determined. Lower expression of ALT, AST in serum were observed in DBT or Orl treated mice (**Supplementary Figure S1**). Meanwhile, less TNF-α and IL-6 cytokines in liver tissues (**Supplementary Figure S2**) were detected compared with mice in the HFD group. Therefore, the results indicated that DBT administration can decrease lipid accumulation and inflammation in liver and serum, and DBT-L exerted a better effect.

### DBT Promotes Thermogenesis in BAT

**FIGURE 3:**
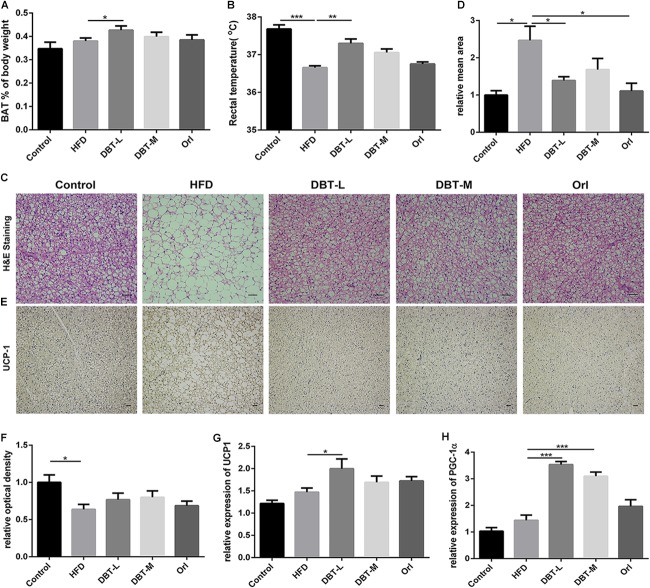
DBT promotes BAT thermogenesis. **(A)** Ratio of BAT to body weight; **(B)** Rectal temperature measured at 0900 h; Haematoxylin and eosin staining of BAT **(C)** and relative mean area **(D)**, scale bar: 20 μm; UCP1 immunohistochemistry of BAT **(E)** and relative optical density **(F)**; qPCR analysis of the mRNA levels of UCP1 **(G)** and PGC-1α **(H)**. Results are presented as mean ± SEM (*n* = 7). ^∗^*p*< 0.05, ^∗∗^*p*< 0.01, ^∗∗∗^*p*< 0.001 compare with HFD group.

To investigate reason of body weight loss, thermogenesis and related regulators were researched. Consistent with the reduction of body weight, BAT of body weight ratio of DBT and Orl treated mice was higher than that in the HFD group, and a statistical significance was observed in the DBT-L group (**Figure 3A**). Less lipids and smaller adipocytes size in the brown adipose tissue were observed than the HFD mice (**Figure 3C**). Besides, the rectal temperature of DBT-L treated mice were higher than that in the HFD group significantly (**Figure 3B**), which indicates these mice could generate higher heat. The rectal temperature of DBT-M and Orl group mice were also higher than that of HFD mice, though there was not statistical significance (**Figure 3B**). UCP1, a classical BAT marker gene, was increased remarkably in DBT and Orl treated groups by immunohistochemical analysis (**Figure 3D**). Meanwhile, administration of DBT or Orl induced the activation of genes controlling thermogenesis in BAT. The expression levels of UCP1 and transcription factor PGC-1α, which were critical transcriptional co-activators of nuclear receptors inducing UCP1 expression (Puigserver et al., 1998), were also increased in the DBT treatment groups, especially in the DBT-L group (**Figures 3E,F**). The results revealed that DBT administration could promote thermogenesis in BAT.

### DBT Induces Browning of Epididymal WAT

**FIGURE 4:**
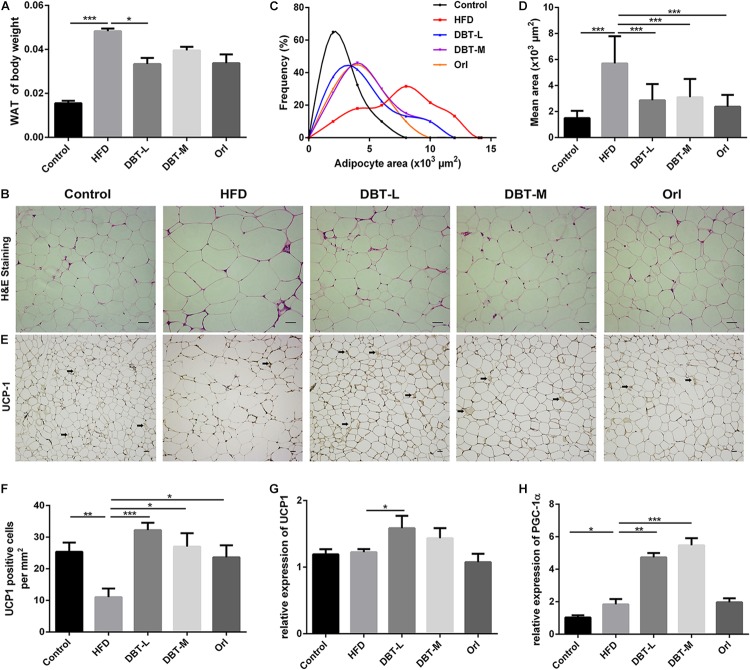
DBT induces epididymal WAT browning. **(A)** Ratio of epididymal WAT to body weight; **(B)** Haematoxylin and eosin staining of epididymal WAT, scale bar: 20 μm; **(C)** Distribution of area and **(D)** mean area of adipocytes of epididymal WAT; **(E)** UCP1 immunohistochemical of epididymal WAT; **(F)** Numbers of UCP1 positive adipocytes in the images; qPCR analysis of the mRNA levels of UCP1 **(G)** and PGC-1α **(H)**. Results are presented as mean ± SEM (*n* = 7). ^∗^*p*< 0.05, ^∗∗^*p*< 0.01, ^∗∗∗^*p*< 0.001 compare with HFD group.

As activated BAT contributed to body weight loss in DBT treated groups, we conjectured whether WAT involves in the phenomenon. As we expected, the epididymal adipose tissue mass significantly reduced in the DBT-M and Orl treated groups than that in the HFD group (**Figure 4A**). Meanwhile, H&E staining analysis indicated that adipocyte size in DBT or Orl treated groups was also smaller, which was correlated with weight loss (**Figures 4B–D**). Moreover, immunohistochemical results manifested that UCP1 expression in DBT and Orl groups were up-regulated strikingly, especially in DBT-L group (**Figures 4E,F**). The BAT marker UCP1 and PGC-1α in epididymal WAT were also significantly increased in the DBT-L groups, rather than that in Orl group (**Figures 4G,H**). All these results demonstrated DBT treatment could promote browning of epididymal WAT.

### DBT Increases pAMPK in WAT

**FIGURE 5:**
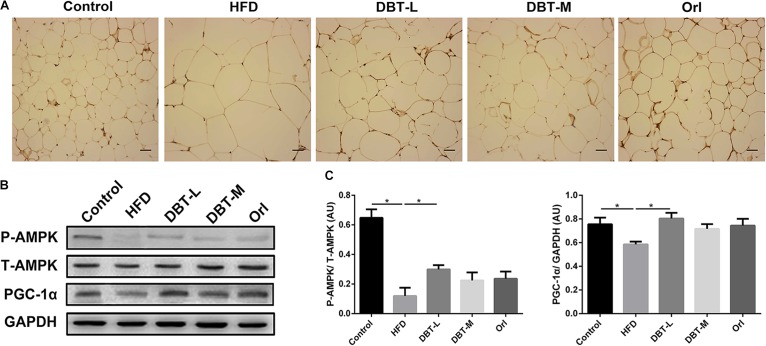
DBT improves phosphorylation of AMPK in WAT. **(A)** Immunohistochemical of pAMPK in epididymal WAT; **(B)** The protein expression of phosphorylation or total AMPK and PGC-1α with GAPDH as control; **(C)** Quantification of P-AMPK and PGC-1α protein expression. Results are presented as mean ± SEM (*n* = 7). ^∗^*p*< 0.05 compare with HFD group.

AMPK plays an important role in energy metabolism (Kahn et al., 2005; Lage et al., 2008). Activation of AMPK induces expression of PGC-1α (Suwa et al., 2003), which regulates thermogenesis by inducing the expression of UCP1 (Wu J. et al., 2013). Based on the above results, we hypothesized that DBT treatment for obesity might be effected by AMPK activation. Therefore, we explored the effects of DBT on the AMPK signaling pathway. It was obvious that phosphorylation of AMPK in DBT treated groups were promoted compared with the HFD group (**Figure 5A**). Besides, protein level of PGC-1α increased in the DBT and Orl treated groups (**Figures 5B,C**). All the results suggested DBT administration could improve energy metabolism by activating AMPK pathway.

## Discussion

**FIGURE 6:**
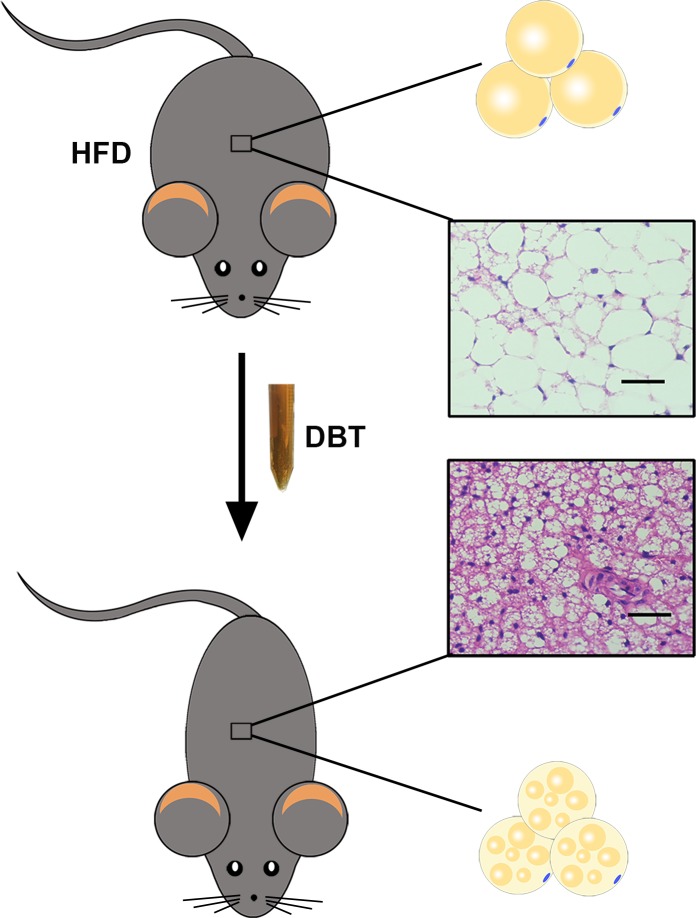
DBT treatment reduced adipose tissue weight and the size of adipocytes by activating thermogenesis.

Obesity is a complicated process determined by interactions between genetic variation in physiology and the environment. Currently, knockout and transgenic animals have also been used to mimic human obesity. However, in modern society, human obesity involves excessive food intake and lack of exercise, and mice with high-fat diet are most close to the obese state in human beings (Niu et al., 2010). Therefore, in this study, we evaluated the effects of DBT in mice with a high-fat diet and found that administration of DBT decreased body weight, the levels of TG, TC, and serum glucose in high-fat diet-induced obese mice. Lipid droplets deposition in liver and adipose tissue were also reduced (**Figure 6**). Meanwhile, DBT could reduce weight of white adipose tissue, size of adipocytes, and elevated rectal temperature by phosphorylation of AMPK to promote expression of UCP1 and PGC-1α. It is worth mentioning that DBT-L exert better anti-obesity effects than DBT-M. The reason may be that DBT-M group metabolizes more byproducts, which affects the active substances and its pharmacological effects. Therefore, the anti-obesity mechanism of DBT may be mainly attributed to thermogenesis through phosphorylation of AMPK.

Obesity is often accompanied with chronic inflammation (Sun and Karin, 2012), which is believed to contribute to the development of obesity-associated metabolic diseases (Eckel et al., 2011). Cytokines, such as TNF-α and IL-6 play important roles in the systemic inflammatory response (Fernandez-Sanchez et al., 2011). Our results indicated DBT decreased the expression levels of TNF-α and IL-6 in adipose tissue and liver (**Supplementary Figures S2**, **S3**). Meanwhile, ALT and AST levels, the biomarkers for liver inflammation (Karmen et al., 1955), also reduced in serum of DBT treated groups compared with the model group. It was indicated that DBT could alleviate HFD-induced inflammation.

There are two specialized types of adipose tissue, WAT and BAT. WAT is characterized by 90% of the volume containing single unilocular lipid droplet and squeezed nucleus, whereas BAT composes of multilocular lipid droplets and numerous large mitochondria (Cinti, 2012; Zhang et al., 2014). Mitochondria in BAT are signed with the high level of uncoupling protein 1 (UCP1), which could uncouple respiration and dissipates chemical energy as heat to prevent obesity (Frontini et al., 2007). Therefore, white adipocytes and brown adipocytes are different in morphology and physiology- white adipocytes store energy to regulate diverse activities, whereas brown adipocytes increase energy expenditure for thermogenesis (Murano et al., 2009; Vitali et al., 2012). Similar to brown adipocytes, beige adipocytes also express more UCP1 in WAT in response to cold exposure or activation of β-adrenergic receptors (Harms and Seale, 2013). And the emergency of beige adipocytes in white fat depots is named browning of white adipose tissue (Harms and Seale, 2013). In recent years, strategies that regulate WAT browning remain an alternative approach for increasing energy expenditure and decreasing obesity (Tseng et al., 2010; Harms and Seale, 2013; Chen Y. et al., 2017). Herbal medicines are explored to combat obesity by promoting thermogenesis (Zhang et al., 2014; Chen G. et al., 2017). In our study, administration of DBT could decrease the size of adipocyte in BAT and WAT in HFD-induced obese mice, and increase the expression of transcription factor UCP1 and PGC-1α (**Figures 3**, **4**), which indicates that DBT promotes BAT thermogenesis and the browning of WAT. It is interesting to compare the effects of DBT and Orl. It has been pharmacologically revealed that orlistat, a lipase inhibitor approved by FDA for long-term anti-obesity, decreased absorption of dietary fat effects (Heck et al., 2000), which used as a positive control in our study. Based on the results, DBT and Orl could decrease body weight and liver lipid accumulation to prevent obesity. However, thermogenesis-related cytokines were not significantly increased in Orl treatment group, which may be attributed to the different action mechanism of anti-obesity.

AMPK plays a critical role in regulating fatty acid metabolism and energy homeostasis (Kahn et al., 2005; Lage et al., 2008; Wu et al., 2018). AMPK is expressed in liver, adipose tissue and several key hypothalamic nuclei (Lage et al., 2008; Lopez et al., 2008). It was reported that AMPK activation could inhibit cholesterol and fatty acid synthesis in liver, reduce lipolysis in adipose tissue, as well as decrease fatty acid synthesis in hypothalamus (Lage et al., 2008). Berberine, a natural compound, has been shown to promote thermogenesis by activating AMPK pathway in white and brown adipose tissue (Zhang et al., 2014). Several studies have suggested that AMPK activation negatively regulates white adipocyte differentiation and positively regulated brown adipocyte adipogenesis (Yang et al., 2016). PGC-1α is a critical player in the brown adipose tissue metabolism by increasing mitochondrial biogenesis and UCP1 expression (Puigserver et al., 1998; Jager et al., 2007). AMPK activation could induce PGC-1α expression to regulate fatty acid oxidation and also promote energy expenditure in subcutaneous WAT (Vila-Bedmar et al., 2010; Gaidhu et al., 2011). Meanwhile, it was reported that AMPK signaling pathway was affected in HFD-induced obese mice (Martin et al., 2006; Tanaka et al., 2007), as well as in the db/db mice (Martin et al., 2006). Thus, it was suggested that targeting AMPK might be a potential therapy for obesity (Lopez et al., 2008). In our study, the expression levels of transcription factor PGC-1α and phosphorylation of AMPK were increased in the mice administrated with DBT, which manifested that the increase of energy metabolism was the main action mechanism of DBT. At the mean time, DBT may also influence other signaling pathways, such as BMP4 and Smad1 signaling pathway, so we tested the expression level of BMP4 and Smad1 in the epididymal WAT, and found that BMP4 and Smad1 were decreased in the HFD group, but increased in the DBT and Orl treated groups (**Supplementary Figure S4**). Therefore, DBT may also influence the BMP4 and Smad1 signaling pathway, and we will study its mechanism of action more comprehensively in the future.

## Conclusion

This study firstly suggested that DBT administration exhibited anti-obesity effect, especially at the DBT-L dose. DBT could also inhibit lipid accumulation and enhanced white adipose tissue browning through phosphorylation AMPK in HFD-induced obese mice. As a consequence, DBT might be a potential therapy against obesity. However, further investigation is needed for better understanding of its effects on obesity. It will be of interest to clarify whether DBT improves metabolic disorders, and whether DBT affects other signaling pathways.

## Author Contributions

LZ and RC designed the experiments. LZ and XZ performed the research and analyzed the results. LZ wrote the draft manuscript. XY and YZ revised and approved the submitted version.

## Conflict of Interest Statement

The authors declare that the research was conducted in the absence of any commercial or financial relationships that could be construed as a potential conflict of interest.

## Supplementary Material

FIGURE S1 DBT decreases aminotransferase in serum. **(A)** alanine aminotransferase (ALT) level; **(B)** aspartate aminotransferase (AST) level. Results are presented as mean ± SEM (*n* = 7). ^∗∗^*p*< 0.01, ^∗∗∗^*p*< 0.001 compare with HFD group.

FIGURE S2 DBT reduces inflammatory cytokines in liver. **(A)** TNF-α; **(B)** IL-6. Results are presented as mean ± SEM (*n* = 7). ^∗^*p*< 0.05, ^∗∗^*p*< 0.01, ^∗∗∗^*p*< 0.001 compare with HFD group.

FIGURE S3 DBT suppresses inflammatory cytokines expression in WAT. **(A)** TNF-α; **(B)** IL-6. Results are presented as mean ± SEM (*n* = 7). ^∗^*p* < 0.05, ^∗∗^*p*< 0.01, ^∗∗∗^*p*< 0.001 compare with HFD group.

FIGURE S4 DBT increases cytokines expression in epididymal WAT. **(A)** BMP4; **(B)** Smad1. Results are presented as mean ± SEM (*n* = 7). ^∗^*p* < 0.05, ^∗∗∗^*p* < 0.001 compare with HFD group.

## Abbreviations

ALT: alanine aminotransferase
AMPK: AMP-activated protein kinase
AST: aspartate aminotransferase
BAT: brown adipose tissue
BMP4: bone morphogenetic protein 4
CHREBP: carbohydrate response element binding protein
Fas: fatty acid synthase
FBG: fasting blood glucose
HFD: high fat diet
IL-6: interleukin
OGTT: oral glucose tolerance
PGC-1α: peroxisome proliferator-activated receptor-gamma coactivator-1alpha
SREBP-1: sterol response element binding protein 1
TC: total cholesterol
TG: triacylglycerol
TNF-α: tumor necrosis factor-α
UCP1: uncoupling protein-1
WAT: white adipose tissue
