# Supplementary figures and images for: The Protective Effects of Danggui-Baizhu-Tang on High-Fat Diet-Induced Obesity in Mice by Activating Thermogenesis
Source: Front Pharmacol. 2018 Sep 5;9:1019. doi: 10.3389/fphar.2018.01019 (PMC6143821; doi:10.3389/fphar.2018.01019)

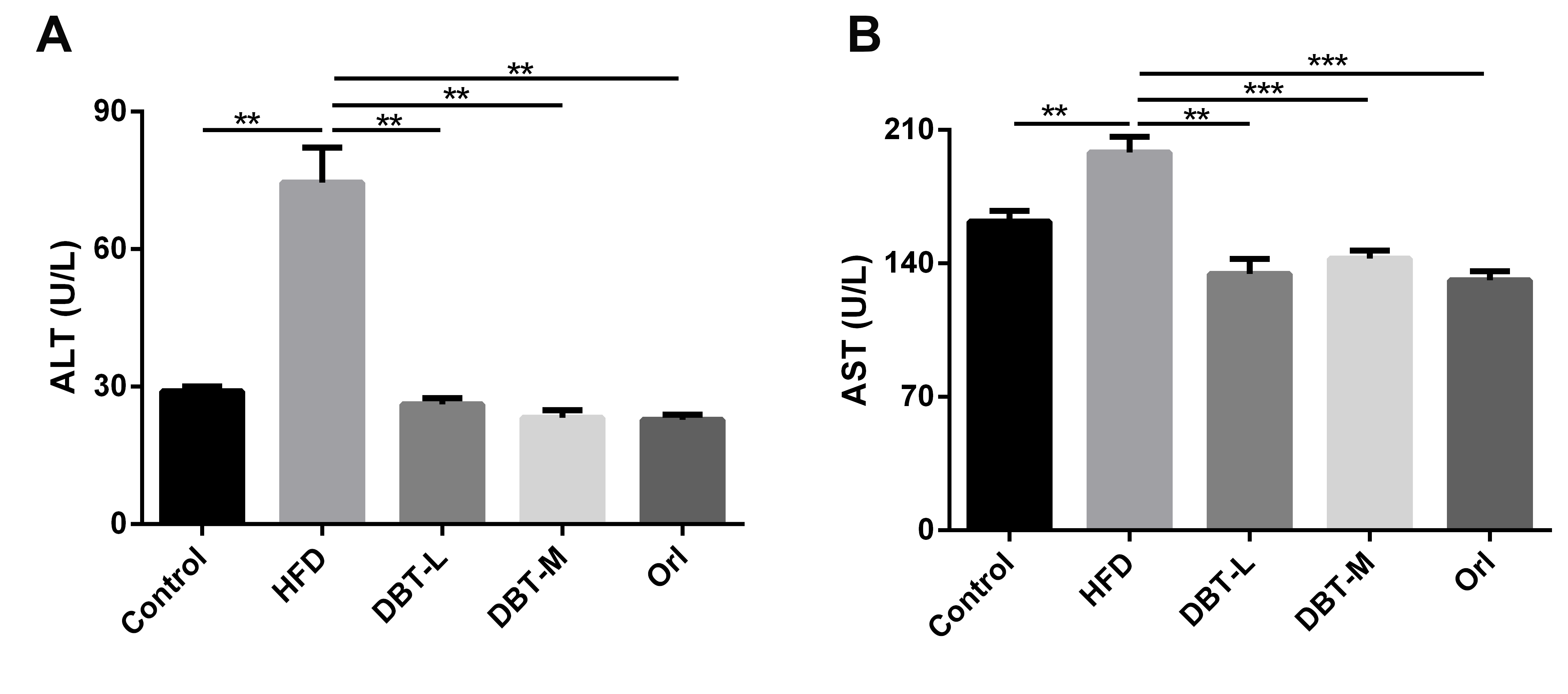

Supplement: FIGURE S1 — DBT decreases aminotransferase in serum. (A) alanine aminotransferase (ALT) level; (B) aspartate aminotransferase (AST) level. Results are presented as mean ± SEM (n = 7). ∗∗p< 0.01, ∗∗∗p< 0.001 compare with HFD group. [file Image_1.TIF]

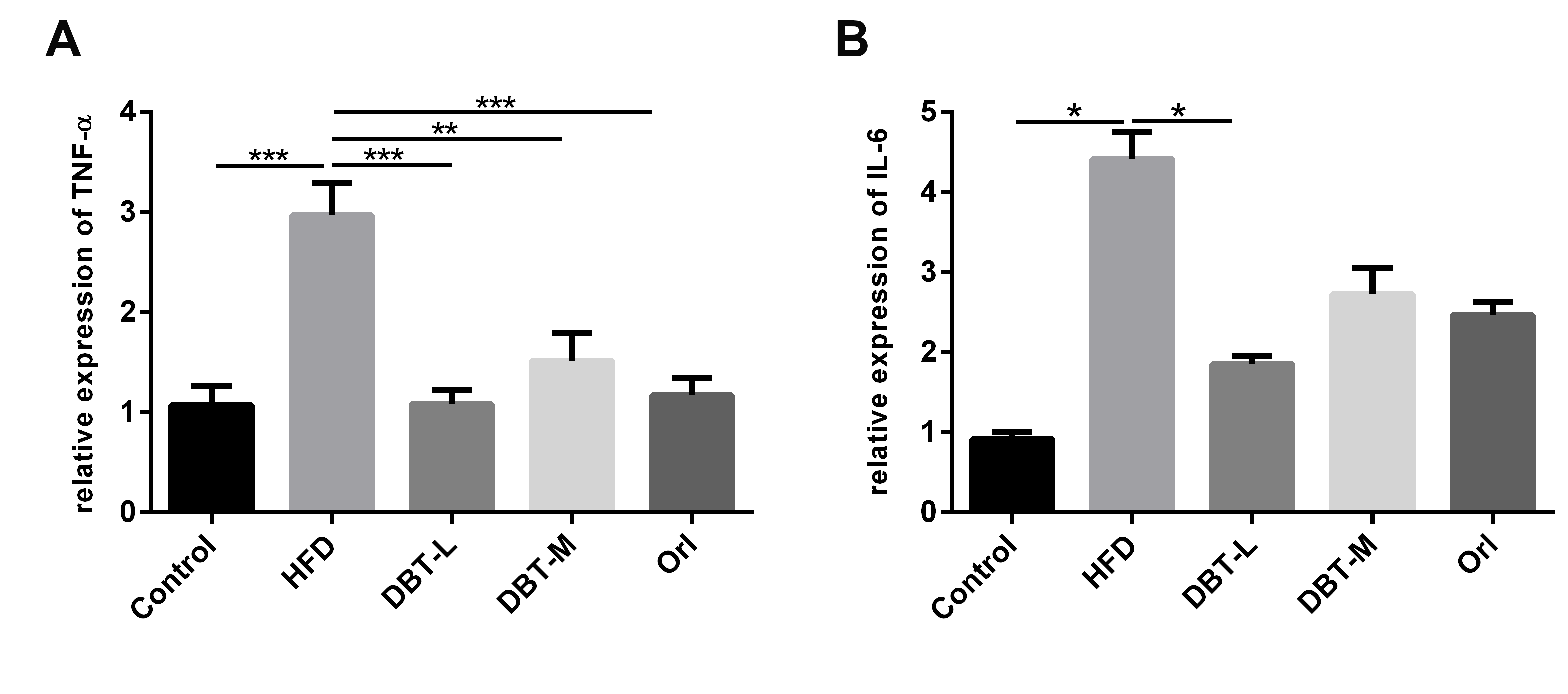

Supplement: FIGURE S2 — DBT reduces inflammatory cytokines in liver. (A) TNF-α; (B) IL-6. Results are presented as mean ± SEM (n = 7). ∗p< 0.05, ∗∗p< 0.01, ∗∗∗p< 0.001 compare with HFD group. [file Image_2.TIF]

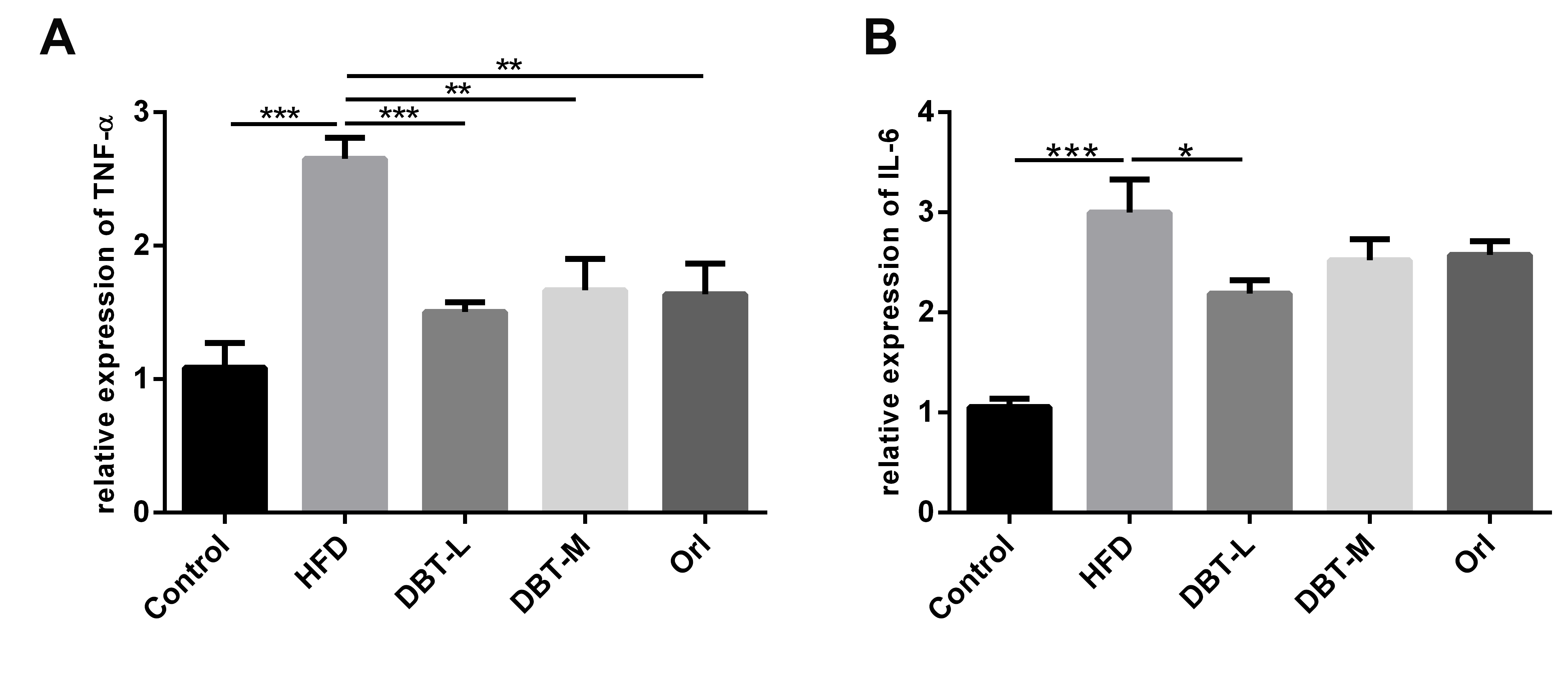

Supplement: FIGURE S3 — DBT suppresses inflammatory cytokines expression in WAT. (A) TNF-α; (B) IL-6. Results are presented as mean ± SEM (n = 7). ∗p < 0.05, ∗∗p< 0.01, ∗∗∗p< 0.001 compare with HFD group. [file Image_3.TIF]

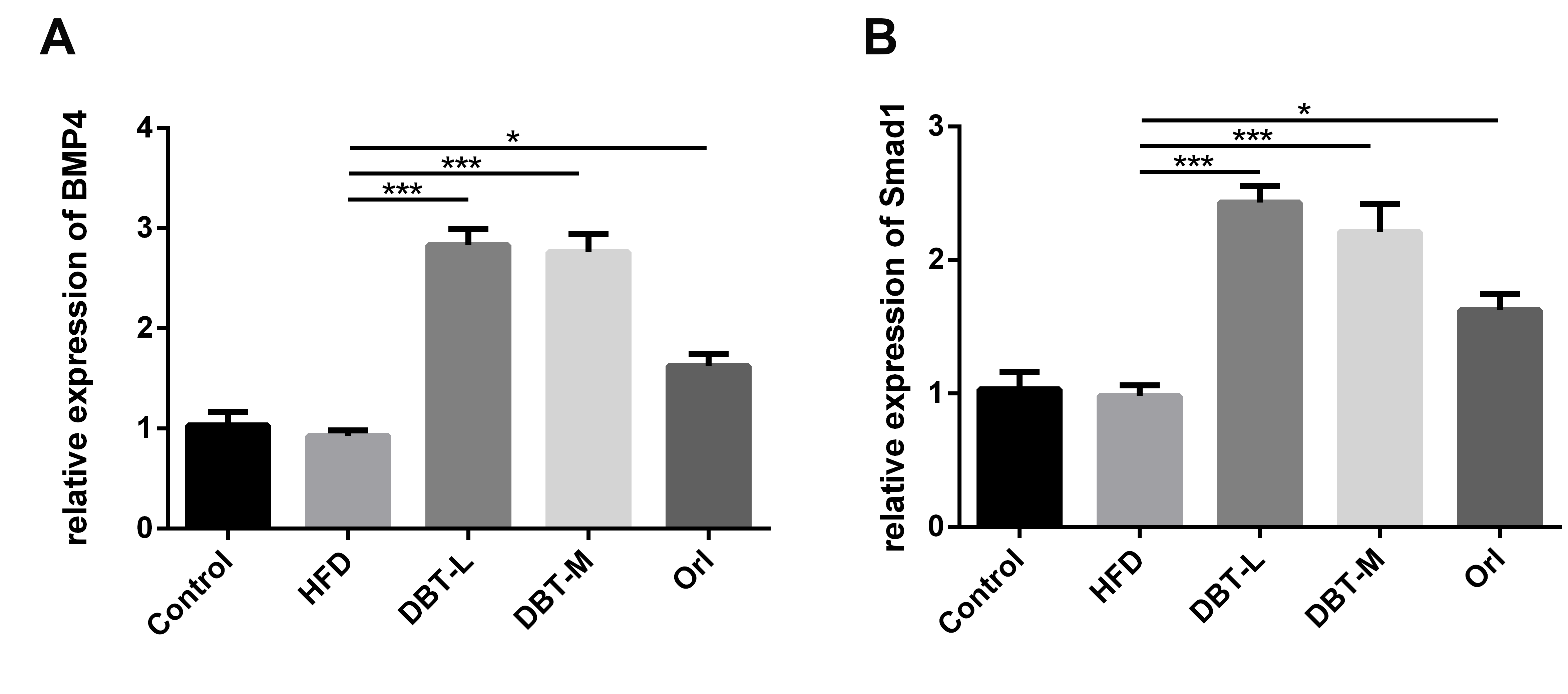

Supplement: FIGURE S4 — DBT increases cytokines expression in epididymal WAT. (A) BMP4; (B) Smad1. Results are presented as mean ± SEM (n = 7). ∗p < 0.05, ∗∗∗p < 0.001 compare with HFD group. [file Image_4.TIF]
