# Supplementary material for: The Protective Effects of Danggui-Baizhu-Tang on High-Fat Diet-Induced Obesity in Mice by Activating Thermogenesis
Source: Front Pharmacol. 2018 Sep 5;9:1019. doi: 10.3389/fphar.2018.01019 (PMC6143821; doi:10.3389/fphar.2018.01019)
Supplement: Supplementary file 5 [file Table_1.pdf]

Supplementary Table 1
Main Components and Content Assay of Danggui-Baizhu-Tang

Chinese herb	Content assay	Pharmacopoeia standard	Test result
Poria	Pachyman	≥ 0.418%	0.8%
Radix Angelicae sinensis	Volatile oil	≥ 0.4%	0.6%
	Ferulic acid	≥0.05%	0.2%
Rhizoma Atractylodis Macrocephalae	Atractylenolide III	≥0.01%	0.03%
Fructus Aurantii Immaturus	Synephrine	≥0.3%	1.08%
